# Infant Stool Color Card Screening Helps Reduce the Hospitalization Rate and Mortality of Biliary Atresia: A 14-Year Nationwide Cohort Study in Taiwan

**DOI:** 10.1097/MD.0000000000003166

**Published:** 2016-03-25

**Authors:** Min Lee, Solomon Chih-Cheng Chen, Hsin-Yi Yang, Jui-Hua Huang, Chun-Yan Yeung, Hung-Chang Lee

**Affiliations:** From the Department of Pediatrics (ML), Taipei City Hospital, Zhongxiao Branch, Taipei City; Department of Medical Research (SC-CC, H-YY, J-HH), Ditmanson Medical Foundation Chia-Yi Christian Hospital, Chiayi City; Department of Pediatrics (SC-CC), School of Medicine, Taipei Medical University, Taipei; Department of Pediatrics (C-YY), MacKay Children's Hospital, Taipei City; and MacKay Memorial Hospital (H-CL), Hsinchu Branch, Hsinchu City, Taiwan.

## Abstract

Biliary atresia (BA) is a significant liver disease in children. Since 2004, Taiwan has implemented a national screening program that uses an infant stool color card (SCC) for the early detection of BA. The purpose of this study was to examine the outcomes of BA cases before and after the launch of this screening program. The objectives of this study were to evaluate the rates of hospitalization, liver transplantation (LT), and mortality of BA cases before and after the program, and to examine the association between the hospitalization rate and survival outcomes.

This was a population-based cohort study. BA cases born during 1997 to 2010 were identified from the Taiwan National Health Insurance Research Database. Sex, birth date, hospitalization date, LT, and death data were collected and analyzed. The hospitalization rate by 2 years of age (Hosp/2yr) was calculated to evaluate its association with the outcomes of LT or death.

Among 513 total BA cases, 457 (89%) underwent the Kasai procedure. Of these, the Hosp/2yr was significantly reduced from 6.0 to 6.9/case in the earlier cohort (1997–2004) to 4.9 to 5.3/case in the later cohort (2005–2010). This hospitalization rate reduction was followed by a reduction in mortality from 26.2% to 15.9% after 2006. The Cox proportional hazards model showed a significant increase in the risk for both LT (hazard ratio [HR] = 1.14, 95% confidence interval [CI] = 1.10–1.18) and death (HR = 1.05, 95% CI = 1.01–1.08) for each additional hospitalization. A multivariate logistic regression model found that cases with a Hosp/2yr >6 times had a significantly higher risk for both LT (adjusted odds ratio [aOR] = 4.35, 95% CI = 2.82–6.73) and death (aOR = 1.75, 95% CI = 1.17–2.62).

The hospitalization and mortality rates of BA cases in Taiwan were significantly and coincidentally reduced after the launch of the SCC screening program. There was a significant association between the hospitalization rate and final outcomes of LT or death. The SCC screening program can help reduce the hospitalization rate and mortality of BA cases and bring great financial benefit.

## INTRODUCTION

Biliary atresia (BA), an idiopathic obliteration of the biliary ductal system, is a common cause of liver transplantation (LT) or death from liver disease in infants or children.^1^ The best outcomes are highly correlated with an early diagnosis and surgical treatment.^1–5^ The Kasai procedure (hepatoportoenterostomy) is the primary surgical therapy for BA.^2^ Increased age at the time of the Kasai procedure may result in a progressive and sustained deleterious effect on the outcomes of BA cases.^3–5^ Therefore, reducing the age at which children undergo the Kasai procedure is an important method to improve the outcomes of BA cases.^2,5–7^ Our previous paper found that the Kasai procedure performed at a younger age may reduce the need for LT. The LT rates were 25.6% and 32.3% in patients who received the Kasai procedure within 60 days or after 60 days of age, respectively.^8^

Because Taiwan has a National Health Insurance system with high (almost 99%) coverage of the entire population, we were able to examine the national inpatient records of BA cases based on the National Health Insurance claims data set. Based on this data set, our previous study found that there was a decreasing trend in age at diagnosis and surgery in BA cases. The mean ages at diagnosis were 57.9, 55.6, and 52.6 days in 1997 to 2001, 2002 to 2006, and 2007 to 2011, respectively. In addition, the proportion of BA cases that received the Kasai procedure within 60 days of age gradually increased from 76% to 81%.^8^

To detect BA cases earlier, Taiwan initiated a universal screening program in 2004 that involves using an infant stool color card (SCC).^9–12^ The SCC is placed within the Child Health Handbook that is provided for every newborn. When infants are brought to hospitals or clinics for vaccinations at the first 2 months of age, their parents or guardians will be asked for the infants’ stool color. If a discolored stool is suspected, the infants are referred to a pediatric gastroenterologist for further workup. Because the SCC screening program is a feasible, cost-effective, and beneficial strategy for BA detection,^12–14^ it has currently been applied regionally or nationally in some countries including Brazil, Japan, Switzerland, Canada, and the Netherlands.^13–18^

The aim of this study was to examine whether the implementation of the SCC screening program can improve BA case outcomes. The objectives of this study were to assess the hospitalization, LT, and mortality rates of BA cases before and after the launch of this screening program, and (2) to examine the association between the hospitalization rate and survival outcomes.

## MATERIALS AND METHODS

### Ethical Approval and Data Resource

The Taiwan National Health Insurance Research Database (NHIRD) is a nationwide, population-based reimbursement database designed for research. The identification numbers of all persons and hospitals in this database were encrypted to be unrecognizable from the original identification numbers. Thus, this study represents de-identified secondary data. The Institutional Review Board of our institution waived the requirement for written informed consent from the patients and approved this study (CYCH-IRB No. 102023).

### Case Identification and Incidences

Following the criteria of previous studies,^8,19,20^ we identified BA cases on the basis of the International Classification of Diseases, Ninth Revision, Clinical Modification (ICD-9-CM) code for BA (751.61) plus either the Kasai procedure (ICD-9-CM code 51.37) or LT (ICD-9-CM code 50.5) from Taiwan's NHIRD during 1997 to 2010. We collected and analyzed data on the sex, birth date, and date of each hospitalization and operation from the database. The BA cases from every 2 years were grouped into one period, resulting in a total of 7 periods. We also divided all BA cases into 2 birth cohorts: an earlier cohort (1997–2004) and a later cohort (2005–2010). The frequency and incidences of hospitalization were calculated and compared among the 7 periods and 2 cohorts.

### Statistical Analysis

The data were analyzed by the 2-tailed Student *t* test for continuous variables with a normal distribution, the Mann–Whitney *U* test for continuous variables without a normal distribution, and the χ^2^ test for categorical variables. A Z-test based on the Poisson regression was used to examine the incidences among 7 birth cohorts. Trends in the incidence of BA patients were analyzed by the Cochrane-Armitage trend test. We used a Cox proportional hazards regression to estimate the relative risks for survival outcome (LT, death, and LT/death) among BA patients. The analyses were adjusted for sex, calendar year of birth (1997–2004 or 2005–2010), and age at the Kasai procedure (>60 or ≤60 days). A receiver-operating characteristic (ROC) curve was plotted to determine the optimal cutoff values for the survival outcome (LT, death, and LT/death) based on the number of hospitalizations per case until 2years of age (Hosp/2yr). The sensitivity and specificity of the cutoff values for survival outcomes in BA patients were calculated. A multivariate logistic regression analysis was further performed to identify the risk factors for LT, death, and either LT/death in BA patients. The sex, birth cohort (1997–2004 or 2005–2010), age at the Kasai procedure (>60 or ≤60 days), and Hosp/2yr (>6 or ≤6 times) were entered together into a multivariate logistic regression analysis. All of the statistical procedures were performed using SPSS for Windows version 21.0 (SPSS; IBM Corporation, Somers, NY), and a *P* ≤ 0.05 or less was considered statistically significant. The ROC curve computing and graphics were performed using R, version 3.2.1, with the Epi packages.

## RESULTS

### The Incidences of BA Cases

There were a total of 513 BA cases identified between 1997 and 2010. The incidence of BA ranged between 0.12 and 0.19 per 1000 live births, and did not display a specific trend during the observational period (*P* *=* 0.682). Among the BA cases, 457 (89%) had received the Kasai procedure. The operation rate did not change significantly during the study period (*P* = 0.904). Approximately one-fourth to one-third of BA cases underwent the Kasai procedure after the age of 60 days; this proportion was without significant trend (*P* = 0.251).

### Trends in the Hospitalization Rate and Length of Stay

The average and standard deviation of operation age, the proportion of cases undergoing the operation after 60 days of age, the average Hosp/2yr, and the length of stay (LOS) per hospitalization are summarized in Table 1. The average Hosp/2yr was 4.9 to 6.9 times, and it was significantly reduced after 2004, displaying a decrease from 6.0 to 6.9 times in the earlier cohort (1997–2004) to 4.9 to 5.3 times in the later cohort (2005–2010). However, the LOS of each hospitalization was approximately 76 and 88 days for the earlier and later cohorts, respectively, which did not differ significantly (*P* = 0.432).

**TABLE 1:**
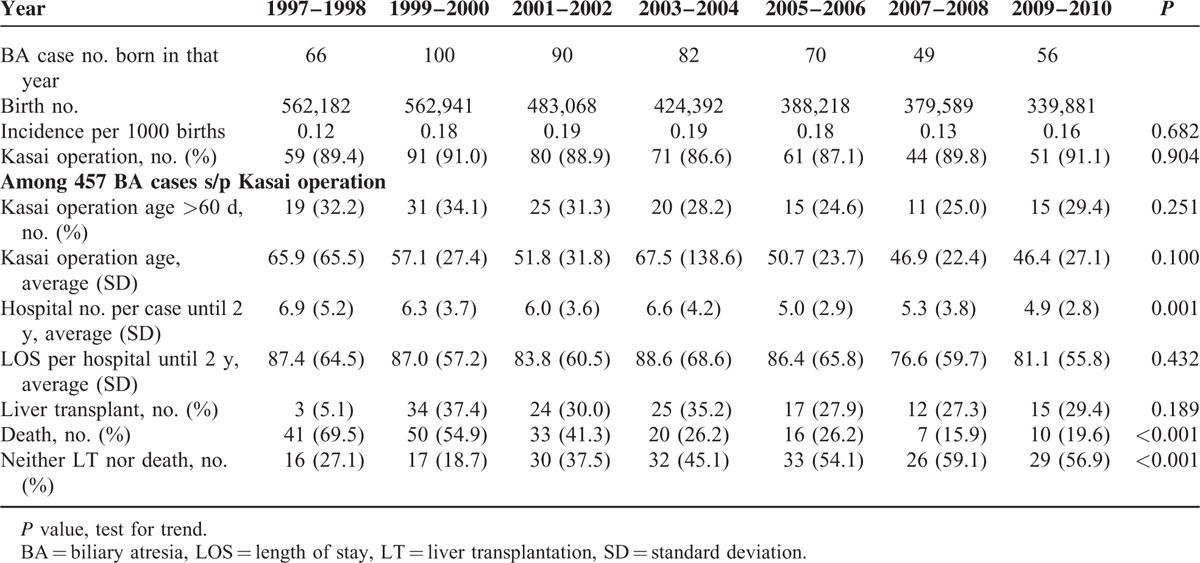
The Case Number, Incidence, Hospitalization, Length of Stay, Death, and Liver Transplant Rates of Biliary Atresia Cases From 1997 to 2010

### Trends of Death or LT of BA Cases

The percentage of death decreased from approximately 70% in the early period to <20% in the most recent period (Table 1). A dramatic reduction of mortality was noted after 2006 (decrease from 26.2% to 15.9%). However, the LT rate remained approximately 30% among BA cases with Kasai procedure. The overall percentage of no LT/death consequently increased from 20% to approximately 60% (*P* < 0.001).

### Comparison Between the 2 Birth Cohorts

As shown in Table 2, the later cohort (2005–2010) had significantly fewer hospitalization incidences than the earlier 1997 to 2004 cohort (5.0 vs 6.4, *P* < 0.001). The mortality was also significantly reduced from 47.8% to 21.2% (*P* < 0.001). Therefore, the overall survival rate without LT/death increased from 31.6% to 56.4% (*P* < 0.001).

**TABLE 2:**
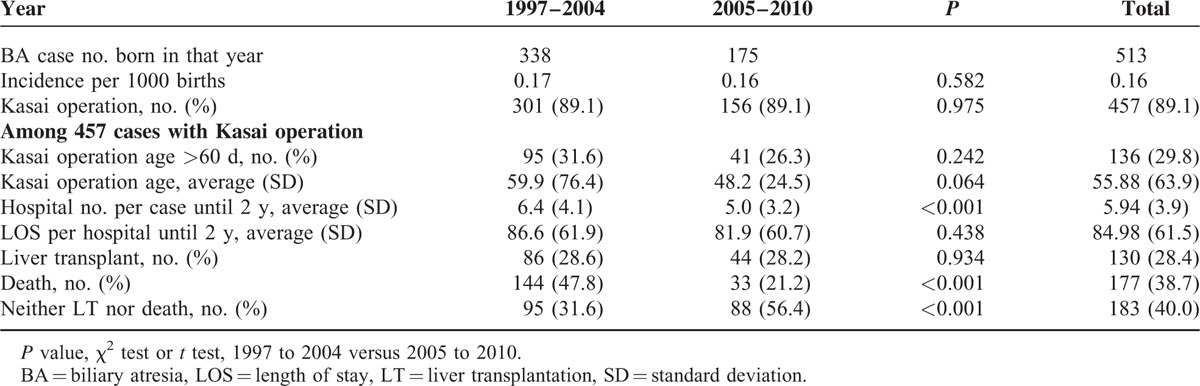
The Case Number, Incidence, Hospitalization, and Length of Stay of Biliary Atresia Cases From 1997 to 2010

### Selection of the Cutoff Value Based on the ROC Curve

**FIGURE 1:**
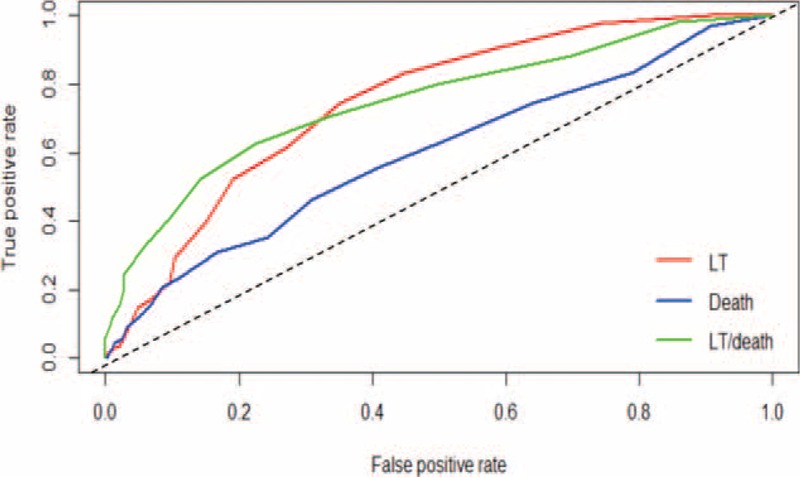
The receiver-operating characteristic curve for predicting the survival outcomes of LT, death, and LT or death in BA cases after the Kasai procedure. BA = biliary atresia, LT = liver transplantation.

Based on the ROC curve, the optimal cutoff value for Hosp/2yr as an indicator for LT was projected to be 5.5 times per case, which yielded a sensitivity of 74.6% and a specificity of 64.8%, with an area under the curve (AUC) of 74.8% (95% confidence interval [CI] = 69.2%–77.9%; Appendix 1 and Figure 1). The AUC, sensitivity, and specificity of Hosp/2yr as an indicator for death were not as good as the values for LT, but they still reached significance (Appendix 1). The combined model increased the specificity for “either LT/death” to 77.6%, with a sensitivity of 62.4%, and the AUC was 74.8% (95% CI: 70.4%–79.3%; Appendix 1 and Figure 1). Based on the ROC results, we chose 6 times as the cutoff value for Hosp/2yr to predict the probability or final outcome.

### Prediction Models for Major Outcomes of BA Cases

The Cox proportional hazards model results in Table 3 shows that there was a 14% increase in risk for LT (hazard ratio [HR] = 1.14, 95% CI = 1.10–1.18) and a 5% increase in risk for death (HR = 1.05, 95% CI = 1.01–1.08) for each additional hospitalization. The multivariate logistic regression model results in Table 4 show that those cases with Hosp/2yr >6 times had significantly higher risk for both LT (adjusted odds ratio [aOR] = 4.35, 95% CI = 2.82–6.73) and death (aOR = 1.75, 95% CI = 1.17–2.62). The later cohort (2005–2010) had a higher risk of LT, but a lower risk of death compared with the earlier cohort (1997–2004). The overall OR of LT/death was significantly reduced (OR = 0.39, 95% CI = 0.25–0.59). Both multivariate regression models found that Hosp/2yr was significantly associated with a higher risk for LT/death after controlling for sex, age at the Kasai procedure, and birth cohort among BA cases.

**TABLE 3:**
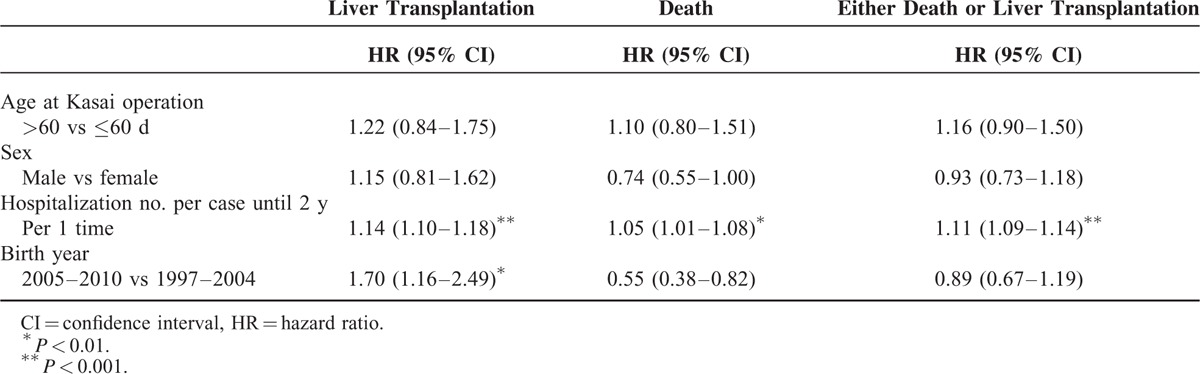
Cox Proportional Hazards Models of Different Outcomes and Related Variables (Age, Sex, Birth Cohort, and Hospitalization Rate)

**TABLE 4:**
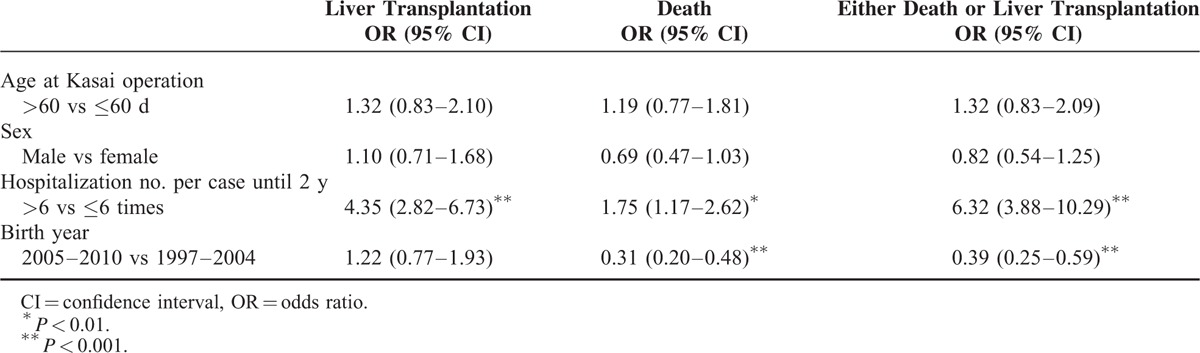
Multivariate Logistic Regression Analysis to Predict Liver Transplantation, Death, and Liver Transplantation or Death of Biliary Atresia Patients

## DISCUSSION

This was a 14-year nationwide population-based cohort study. We found a dramatic reduction in the hospitalization rate of BA cases after 2004, when the SCC program was fully implemented in entire country. Throughout the study period, the mortality rate was also significantly reduced, but the LT rate did not change. However, the overall survival rate, without LT or death, significantly improved. The multivariate analysis found the hospitalization rate was significantly associated with survival outcomes. An additional hospitalization increases the LT and mortality rates by 14% and 5%, respectively. These data may provide valuable information for health professionals and families caring for BA cases.

The SCC screening program has been confirmed to be a sensitive and specific screening method for BA in infants.^9,10^ Universal screening using the SCC can enhance earlier referral, which may lead to timely performance of the Kasai procedure in infants with BA and subsequently improved BA case outcomes.^10,12,17^ For example, the Kasai procedure has improved jaundice-free survival with the native liver at the ages of 3 and 5 years.^10,12^ Therefore, it is reasonable to associate the national implementation of the SCC screening program with a reduction in the hospitalization rate of BA cases.

However, LT and death are the 2 BA case outcomes of most concern. This population-based study further revealed that the hospitalization rate was also significantly associated with the survival outcome of BA cases. The multivariate logistic regression model, which controlled for sex, age at the Kasai procedure, and birth cohort, found that the hospitalization rate was the most important factor associated with LT/death (Table 4). A hospitalization frequency >6 times was associated with a 6.32-fold increased risk of LT or death. Our hypothesis states that more hospitalizations indicate more complications, which can accelerate the development of liver cirrhosis and increase the need for LT. Following the reduction of the hospitalization rate since 2004, we have observed a large reduction in mortality since 2006 (Table 1). Such a time lag is reasonable because death, naturally, occurs later than any other outcome.

Some may argue that it is naïve to credit the improvement of final outcomes to the SCC screening program alone. Although previous studies have documented the benefits of the SCC screening program on increasing 3-month jaundice-free rates,^10,12^ the advantage of the SCC program on final outcomes of BA cases requires more evidence. Notably, this present study has observed a significant association between the hospitalization rate and mortality. As shown in Table 3, one additional hospitalization can increase the risk of LT and death by 14% and 5%, respectively. This result also reminded us that every hospitalization of BA cases is meaningful because of its potential effect on long-term survival outcome. We must adequately care for BA cases in a timely manner and do our best to avoid any unnecessary hospitalizations.

Finally, we evaluated the financial impact of this SCC screening program by comparing the direct medical costs of hospitalizations between 2 eras, before and after 2004. Considering the incidence of BA ranged 0.12 to 0.19 per 1000 births, the annual cost saving of hospitalizations for BA cases among 100,000 live births would be 80 to 120 thousand US dollars (see Appendix 2 for calculation). Specially, this money just implies the direct medical cost of hospitalization, but does not include the costs for outpatient visits and indirect costs like “labor loss of parents,” “transplantation for medical visits,” and “psychological stress.” Moreover, we should notice there is a significant reduction of mortality between these 2 eras, from 47.8% to 21.2% (Table 2). Compared with the small cost of implementation, we think the cost-benefit of this SCC screening program in Taiwan could be tremendous.

## CONCLUSIONS

There was a significant reduction in the hospitalization rate of BA cases in Taiwan, which coincided with the full implementation of the SCC screening program in 2004. The average hospitalization rate before 2 years of age was significantly associated with the probability of LT or death. One additional hospitalization could increase the certain risk of LT and death. Therefore, every hospitalization of BA cases is meaningful because of its potential effect on long-term survival. We must care for BA cases to the best of our abilities and avoid any unnecessary hospitalizations. Finally, we estimated the financial impact and calculated the annual cost-saving for hospitalizations of BA cases among 100,000 live births to be 80 to 120 thousand US dollars. Compared with the small cost of implementation, the cost-benefit of this SCC program could be tremendous.

## LIMITATIONS

There are some limitations to this study. First, the source data set was generated from the claims data of the National Health Insurance system. We were unable to identify the true proportion of infants with BA who were actually detected by infant SCC screening because there was no information regarding this rate. In general, all screening programs may be limited by the ability to determine the real detection rate using this screening tool. Second, the hospitalization rate in one country can be affected by many factors such as the accessibility of health care, the insurance, and payment system. Therefore, the cutoff points of the hospitalization rate to predict the final outcomes would differ among various countries. This should be adjusted based on local data.

## Supplementary Material

Supplemental Digital Content
