# Supplementary material for: Infant Stool Color Card Screening Helps Reduce the Hospitalization Rate and Mortality of Biliary Atresia: A 14-Year Nationwide Cohort Study in Taiwan
Source: Medicine (Baltimore). 2016 Mar 25;95(12):e3166. doi: 10.1097/MD.0000000000003166 (PMC4998398; doi:10.1097/MD.0000000000003166)
Supplement: Supplemental Digital Content [file medi-95-e3166-s001.doc]

**Appendix 1. Table. Receiver operating characteristic analysis showing the estimated optimal cut-off value of hospitalization number in predicting LT, death, either death/LT of biliary atresia cases**

| **Outcomes** | **AUC (95% CI)** | **p-value** | **Cut off** | **Sensitivity** | **Specificity** |
| --- | --- | --- | --- | --- | --- |
| LT | 74.8% (70.2%-79.5%) | <0.001 | ≥ 5.5 | 74.6% | 64.8% |
| Death | 59.6% (54.2%-65.0%) | 0.027 | ≥ 6.5 | 46.3% | 68.9% |
| Either LT or Death | 74.8% (70.4%-79.3%) | <0.001 | ≥ 5.5 | 62.4% | 77.6% |

LT = liver transplantation

BA = biliary atresia

**Appendix 2. Estimation of the financial impact -**

**The saving of medical cost for hospitalizations of BA cases before and after 2004.**

According to Table 1&2: we can get some information between two eras as below:

- Difference of hosp. number per case: 6.4x vs. 5.0x (difference = 1.4x, p < 0.001)
- Difference of mortality rate: 47.8% vs. 21.2% (p < 0.001)
- The incidence of BA ranged between 0.12-0.19 per 1,000 births (Table 1).
- According to the original study dataset (NHIRD), the direct medical cost of once hospitalization for BA cases with Kasai operation was mean (SD) = 4810 (4927) in US dollars.

The annual cost saving of hospitalizations of BA cases among 100,000 live birth is

= (live births) x (incidence) x (difference of hosp.) x (cost/hosp.)

= 100,000 births x (0.12-0.19 case/1,000 births) x 1.4 times/case x 4810 (USD$/time)

= 80,808 ~ 127,946 (USD$)
